# Detection of Lipoprotein X (LpX): A challenge in patients with severe hypercholesterolaemia

**DOI:** 10.2478/jomb-2019-0038

**Published:** 2020-09-02

**Authors:** Agnieszka Ćwiklińska, Agnieszka Mickiewicz, Robert Kowalski, Barbara Kortas-Stempak, Agnieszka Kuchta, Krzysztof Mucha, Michał Makowiecki, Anna Gliwińska, Krzysztof Lewandowski, Leszek Pączek, Marcin Fijałkowski, Marcin Gruchała, Maciej Jankowski

**Affiliations:** 1 Medical University of Gdansk, Department of Clinical Chemistry, Gdansk, Poland; 2 Medical University of Gdansk, 1st Department of Cardiology, Gdansk, Poland; 3 Medical University of Gdansk, Hospital Pharmacy of the University Clinical Centre, Gdansk, Poland; 4 Medical University of Warsaw, Transplantation Institute, Department of Immunology, Transplantology and Internal Medicine, Warsaw, Poland; 5 Medical University of Gdansk, Central Laboratory of the University Clinical Centre, Gdansk, Poland; 6 Medical University of Gdansk, Department of Laboratory Medicine, Gdansk, Poland

**Keywords:** lipoprotein X, severe hypercholesterolaemia, cholestasis, hepatobiliary disorders, electrophoresis, lipoprotein X, teška hiperholesterolemija, holestaza, hepatobilijarno oboljenje, elektroforeza

## Abstract

**Background:**

Lipoprotein X (LpX) is an abnormal lipoprotein fraction, which can be detected in patients with severe hypercholesterolaemia and cholestatic liver disease. LpX is composed largely of phospholipid and free cholesterol, with small amounts of triglyceride, cholesteryl ester and protein. There are no widely available methods for direct measurement of LpX in routine laboratory practice. We present the heterogeneity of clinical and laboratory manifestations of the presence of LpX, a phenomenon which hinders LpX detection.

**Methods:**

The study was conducted on a 26-year-old female after liver transplantation (LTx) with severely elevated total cholesterol (TC) of 38 mmol/L and increased cholestatic liver enzymes. TC, free cholesterol (FC), cholesteryl esters (CE), triglycerides, phospholipids, HDL-C, LDL-C, and apolipoproteins AI and B were measured. TC/apoB and FC:CE ratios were calculated. Lipoprotein electrophoresis was performed using a commercially available kit and laboratory-prepared agarose gel.

**Results:**

Commercially available electrophoresis failed to demonstrate the presence of LpX. Laboratory-prepared gel clearly revealed the presence of lipoproteins with γ mobility, characteristic of LpX. The TC/apoB ratio was elevated and the CE level was reduced, confirming the presence of LpX. Regular lipoprotein apheresis was applied as the method of choice in LpX disease and a bridge to reLTx due to chronic liver insufficiency.

**Conclusions:**

The detection of LpX is crucial as it may influence the method of treatment. As routinely available biochemical laboratory tests do not always indicate the presence of LpX, in severe hypercholesterolaemia with cholestasis, any discrepancy between electrophoresis and biochemical tests should raise suspicions of LpX disease.

## Abstract (sr)

**Uvod:**

Lipoprotein X (LpX) je patološka frakcija lipoproteina koja se može naći kod pacijenata sa teškom hiperholesterolemijom i holestatskih oboljenja jetre. LpX se uglavnom sastoji od fosfolipida i slobodnog holesterola, sa malim količinama triglicerida, estara holesterola i proteina. Ne postoje metode za direktno merenje LpX u rutinskoj laboratorijskoj praksi. Ovde je prikazana heterogenost kliničkih i laboratortijskih manifestacija prisustva LpX, fenomena koji ometaju otkrivanje LpX.

**Metode:**

Izučavanje je izvedeno na 26-godišnjoj ženi posle transplantacije jetre (LTx) sa izrazito povećanim ukupnim holesterolom (TC) vrednosti 38 mmol/L i holestatskim jetrenim enzimima, a mereni su i TC, slobodni holesterol (FC), holesteril estri (CE), trigliceridi, fosfolipidi, HDL-C, LDLC i apolipoproteini AI i B. Izračunati su odnosi TC/apoB i FC:CE. Izvedena je elektroforeza apolipoproteina primenom komercijalnog kita i agaroza gela koji je pripremljen u laboratoriji.

**Rezultati:**

Komercijalnom elektroforezom nije bilo moguće utvrditi prisustvo LpX. Primenom laboratorijski pripremljenog gela utvrđeno je prisustvo lipoproteina čija je pokretljivost odgovarala LpX. Odnos TC/apo B je bio povećan, a nivo CE snižen, što je potvrdilo prisustvo LpX. Lipoproteinska afereza je primenjena kao metoda izbora za oboljenja sa LpX i vezu sa relLTx usled hronične insuficijencije jetre.

**Zaključak:**

Za otkrivanje LpX veoma je važan izbor metode. S obzirom da rutinski raspoloživi biohemijski laboratorijski testovi uvek ne ukazuju na prisustvo LpX u teške hiperholesterolemije sa holestazom, bilo koja razlika između eketroforeze i biohemijskih testova treba da ukaže na sumnju da postoji LpX oboljenje.

## Introduction

Severe hypercholesterolaemia with a total cholesterol (TC) concentration above 25 mmol/L (∼1000 mg/dL) is an extremely rare condition. The most widely recognised cause is homozygous familial hypercholesterolaemia (HoFH) presenting with an increased level of LDL-cholesterol (LDL-C) and accelerated advanced cardiovascular disease (CVD) [1]. However, severe hypercholesterolaemia may also be unrelated to increased LDL-C, resulting instead from the presence of an abnormal lipoprotein fractionlipoprotein X (LpX) [2] [3] [4] [5]. LpX is most frequently detected in patients with cholestatic liver disease [6] [7] as well as in those with lecithin:cholesterol acyltransferase (LCAT) deficiency, hepatic lipase (HL) deficiency, and after intravenous fat emulsion infusion [6]. The LpX was not proved to result in coronary artery disease development [8] [9]. Moreover, the antioxidative properties of LpX may reduce LDL atherogenicity [8]. On the other hand, it has been shown that LpX may be associated with hyperviscosity syndrome and lead to renal disease in cases of LCAT deficiency [10].

LpX takes the form of a spherical particle with a diameter exceeding 30 nm, composed largely of phospholipid (PL) and free cholesterol (FC), with small amounts of triglyceride (TG), cholesteryl ester (CE) and protein, but containing no apolipoprotein B (apoB). There are no widely available methods for direct measurement of LpX in routine laboratory practice [11]. However, due to its chemical composition, patients in whom this lipoprotein is present in serum may exhibit elevated TC/apoB and reduced CE level. The presence of LpX can also be detected using electrophoretic techniques. LpX displays γ mobility or moves towards a cathode on agarose gel [11] [12]. Unfortunately, these analyses are not routinely performed in laboratories.

In this paper, we present the heterogeneity of clinical and laboratory manifestations of the presence of lipoprotein X (LpX), a phenomenon which can hinder LpX detection and diagnosis. We also present the potential for the use of different biochemical tests and agarose electrophoretic techniques to detect LpX, taking into account our experiences and reports from the literature.

## Materials and Methods

The study was undertaken on a 26-year-old female with severely elevated TC above 25 mmol/L and increased cholestatic liver enzymes.

A peripheral blood sample was taken using commercially available test tubes following overnight fasting. The following biochemical parameters were assessed: lipid parameters (TC, TG, HDL-C, LDL-C, FC, CE, and PL), apolipoproteins AI and B, liver parameters (AST, ALT, GGT, ALP, and bilirubin), glucose, creatinine and INR (Table 1). For each analyte, the assay was performed according to the manufacturer's instructions.

**Table 1:** Methodology of biochemical parameters

Parameter	Method	Reagent manufacturer	Analyser
Apolipoprotein AI (apo AI)	immunonephelometry	Siemens Healthcare GmbH (Germany)	BN II System
Apolipoprotein B (apo B)	immunonephelometry	Siemens Healthcare GmbH (Germany)	BN II System
Alanine aminotransferase (ALT)	NADH (without P-5’-P)	Abbott Laboratories (USA)	Architect c8000
Alkaline phosphatase (ALP)	p-nitrophenol	Abbott Laboratories (USA)	Architect c8000
Aspartate aminotransferase (AST)	NADH (without P-5’-P)	Abbott Laboratories (USA)	Architect c8000
Total bilirubin	diazonium salt	Abbott Laboratories (USA)	Architect c8000
Cholesteryl ester (CE)	calculated as the difference between TC and FC		
Creatinine	enzymatic	Abbott Laboratories (USA)	Architect c8000
Free cholesterol (FC)	CHOD-PAP	Greiner Laboratories GmbH (Germany)	MultiScan Go
Gamma-Glutamyl Transferase (GGT)	L-Gamma-glutamyl-3- carboxy-4-nitroanilide	Abbott Laboratories (USA)	Architect c8000
Glucose	hexokinase/G6PD	Abbott Laboratories (USA)	Architect c8000
HDL-cholesterol (HDL-C)	direct (Accelerator Selective Detergent)	Abbott Laboratories (USA)	Architect c8000
INR	coagulometric	Siemens Healthcare GmbH (Germany)	BCS XP System
LDL-cholesterol (LDL-C)	calculated from the Friedewald formula		
Sodium	Indirect ISE	Abbott Laboratories (USA)	Architect c8000
Phospholipid (PL)	choline oxidase-DAOS	Wako Pure Chemical Industries (Japan)	MultiScan Go
Total cholesterol (TC)	CHOD-PAP	Abbott Laboratories (USA)	Architect c8000
Triglyceride (TG)	GPO	Abbott Laboratories (USA)	Architect c8000

Lipoprotein electrophoresis was performed using a commercially available electrophoresis kit (Hydragel Lipo+Lp(a), Sebia, France), and with the use of laboratory-prepared agarose gel electrophoresis. The electrophoresis using the commercially available kit was carried out according to the manufacturer's instructions. The laboratory-prepared electro phoresis was performed as follows: aliquots of 10 mL of serum were loaded on agarose gel (0.75% (w/v)) and electrophoresed for 75 minutes at 160 V using a 100 mmol/L Tris-barbital buffer, pH 9.3. After separation, the lipoproteins were visualized by staining with Sudan Black B (1%, (w/v)).

## Results

A 26-year-old female patient was admitted to the Cardiology Department of the Medical University of Gdansk with a diagnosis of severe hypercholesterolaemia complicated by ischaemic central retinal vein occlusion, with vision loss. Physical examination revealed signs of jaundice. Corneal arcus and xanthomas were not present. Checking for hypercholesterolaemia and premature atherosclerotic disease in the family history yielded negative results. The patient's medical history revealed diabetes type 1 and autoimmune hepatitis, diagnosed at the age of 14. At that time, the TC level remained within the normal range (4 mmol/L). Rapidly progressing hepatic failure had resulted in a liver transplantation (LTx) at the age of 16. However, following 6 years of stability, a gradual deterioration of liver function and an increase in lipid parameters were observed. A liver biopsy revealed the rejection of the donor liver. Prednisone (10 mg daily) and cyclosporine (300 mg daily) were administered, with a good response. Nevertheless, a continued increase in TC was observed, even though a rosuvastatin (15 mg daily) and ezetimibe (10 mg daily) were prescribed.

Upon admission to our Centre, laboratory findings showed an exceptionally high concentration of TC (38 mmol/L). The TG level had increased to 6.2 mmol/L; HDL cholesterol (HDL-C) remained within the normal range. Serum biochemical analysis also showed significantly elevated levels of liver enzymes (Table 2). The result of mutational analysis of the LDLR, APOB and PCSK9 genes was negative. Due to a history of elevated liver enzymes, refractoriness to maximally tolerated lipid-lowering medications, and severe hypercholesterolaemia, lipoprotein apheresis (LA) by the lipoprotein filtration technique was initiated. Four courses of LA within an eight-day period reduced TC concentration to 3.8 mmol/L. Sub sequent regular biweekly apheresis treatments resulted in a steady state with pre-apheresis TC and LDL-C levels remaining in the ranges 15.5-20.7 and 5.2-10.4 mmol/L, respectively. However, there was no improvement in serum liver enzymes, which indicated a persistent cholestatic liver dysfunction. Thus, we undertook a series of additional lipid tests.

**Table 2:** Laboratory parameters at admission and following treatment

Parameter	At admission	After treatment	Reference values
TC, mmol/L	38.0	8.7	<4.9
TG, mmol/L	6.2	2.6	<1.7
HDL-C, mmol/L	1.2	0.3	>1.2 (female) >1.0 (male)
LDL-C, mmol/L	not calculated	7.2	<3.0
AST, U/L	216	244	5–34
ALT, U/L	297	196	<55
GGT, U/L	4104	1035	9–36
ALP, U/L	2209	1601	40–150
Total bilirubin, µmol/L	90.3	314.5	1.7 – 20.4
eGFR-MDRD	>60	47	>60
Sodium, mmol/L	131	136	136–145
Glucose, mmol/L	3.9	5.3	<5.6
INR	1.01	1.1	0.9 –1.3

**Figure 1:**
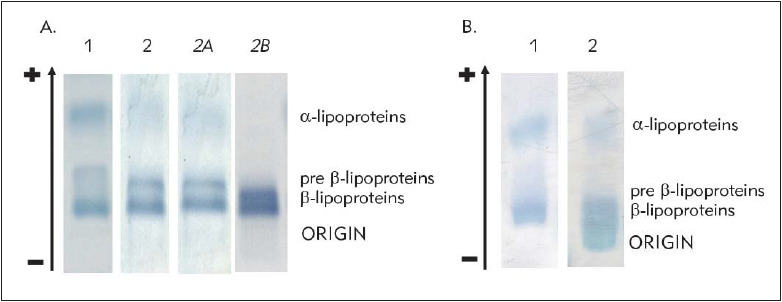
Serum lipoprotein profile obtained using a commercially available electrophoresis kit (A), and laboratory-prepared electrophoresis set (B) 1 – control sample; 2 – sample from the patient. 2A – analysis of a sample obtained from the patient two months after detection of LpX (lipid profile parameters: TC, 17.2 mmol/L; TG, 2.4 mmol/L; HDL-C, 0.6 mmol/L; LDL-C, 15.5 mmol/L); *2B* – analysis of a sample obtained from the patient one year after detection of LpX (lipid profile parameters: TC, 19.2 mmol/L; TG, 7.0 mmol/L; HDL-C, 0.3 mmol/L; LDL-C, not calculated). A subtle fraction with γ mobility was observed only in *2B* analysis

A commercially available agarose electrophoresis set failed to demonstrate the presence of LpX. Intense staining of the band pre-b-mobility areas, characteristic of LDL and VLDL, respectively, was observed, indicating hyperlipoproteinaemia (Hlp) type IIb, and no fraction was observed in the start area (Figure 1A, lane 2). Conversely, a laboratory-prepared agarose electrophoresis set showed a clearly visible fraction with γ mobility and the green tinge characteristic of LpX (Figure 1B, lane 2). The biochemical analysis revealed very high PL and FC levels. The CE level was significantly lower than reference values whereas the TC/apoB ratio was significantly higher (Table 3).

**Table 3:** Lipid and apolipoprotein analysis in the course of LpX detection *according to [11]

Parameter		Reference values
Lipid profile		
TC, mmol/L	21.2	<4.9
TG, mmol/L	4.1	<1.7
HDL-C, mmol/L	0.9	>1.2 (female) >1.0 (male)
LDL-C, mmol/L	18.4	<3.0
Apolipoproteins		
ApoAI, g/L	1.16	
ApoB, g/L	1.92	
Additional lipid analysis		
PL, mmol/L	23.6	
FC, mmol/L	18.8	
CE, mmol/L	2.4	
FC:CE	1 : 0.13	∼ 1 : 2*
TC/apoB, mmol/g	11.0	4.0–7.7 (female)* 3.8–6.3 (male)*

The results of laboratory-prepared electrophoresis and biochemical analysis clearly revealed the presence of LpX in the sample. Its detection focused our efforts on investigating the hepatobiliary causes of severe hypercholesterolaemia. Magnetic resonance revealed stenosis of the biliary tract and a subsequent stent implantation was performed, resulting in slight improvement of cholestatic parameters. Repeated liver biopsies, along with laboratory and imaging tests, led to a diagnosis of chronic liver insufficiency of a complex nature. The patient was placed on the liver transplantation list and referred back to LA as a bridge to re-LTx. After two and a half years of biweekly apheresis therapy, the patient's condition deteriorated rapidly secondary to the development of septic shock followed by sudden death.

## Discussion

We report the heterogeneity of clinical and laboratory manifestations of LpX which can hinder its detection, especially if only routinely available laboratory tests are used.

Differentiation of severe hypercholesterolaemia caused by elevated LDL-C and related to the presence of LpX is important, as it may influence the method of treatment. Although oral lipid-lowering medications are the first-line treatment in hypercholesterolaemia caused by increased LDL-C, they are insufficient and hazardous in LpX disease with coexisting hepatocellular injury. It has been shown that LpX can be safely and efficiently removed by plasma exchange and selective LA, resulting in an improvement of clinical symptoms and normalization of lipid parameters [13]. Regarding the cardiovascular risk in both LDL-C and LpX accumulation, it is clear that increased LDL-C levels lead to accelerated atherosclerosis. Available data on cardiovascular risk in LpX disease are not consistent and further large studies are needed [14] [15]. Nevertheless, it has been proved that LpX accumulation leads to hyperviscosity syndrome. In our described patient, hyperviscosity syndrome resulted in a central retinal vein thrombosis and vision loss [16].

In the case of our patient, LpX detection convinced us that regular bi-weekly apheresis by cascade filtration is the only efficient and safe therapy, as previously described by other authors [17]. We observed a high degree of efficacy of TC removal in LA sessions. Furthermore, the LpX detection directed our attention towards the hepatobiliary causes of hypercholesterolaemia. Prior to that, we had suspected that the patient might have received a liver from a donor with undiagnosed familial hypercholesterolaemia. Nevertheless, further biliary tract stenting failed to improve cholestatic parameters and lipid levels. Liver transplantation remained the only possible method of correcting the underlying liver pathology.

Detection of LpX is also crucial since it can interfere with a number of biochemical tests. For instance, LDL-C calculated using the Friedewald formula is invalid in the presence of LpX, since this equation can be used only if three lipoprotein fractions, VLDL, LDL, and HDL are present in serum. Moreover, LpX can interfere with direct LDL-C assays, the degree of interference being dependent on the method used [18]. There is also evidence that LpX can interfere with the measurements of apolipoprotein E phenotype analysis [19], serum total protein [20], and electrolytes, causing pseudohyponatremia, pseudohypokalemia, and pseudohypochloremia, in cases where indirect ion-selective electrodes are used [21] [22] [23] [24].

Widely available methods for the direct measurement of LpX in routine laboratory practice are lacking [11]. However, there are some laboratory methods capable of providing evidence of the presence of LpX. Agarose electrophoresis is considered one of these [11]. Unfortunately, in our study a commercially available agarose electrophoresis set failed to clearly demonstrate the presence of LpX. Moreover, the presence of LpX manifested itself in different ways in electrophoresis. In the case of our patient, the fraction with mobility lower than β was not detected in two electropherograms, whereas only a subtle smearing band with γ mobility was observed in the third one, performed one year later (Figure 1A, lane 2B). In most studies, co-migration of LpX with β-mobility lipoproteins (LDL) was observed, with eventually subtle reverse migration in the LDL region [14] [22] [23] [25] [26]. Phatlhane et al. [3], in the case of a patient with LpX, observed intense staining in the β area, with minor cathodic migration and a slight green tinge. Less frequently, LpX has been observed at the site of origin, suggesting the presence of chylomicrons [19] [27] or as a smearing lipoprotein band with slow migration [18].

Unlike commercial electrophoresis kits, our laboratory-prepared electrophoresis set clearly demonstrated an additional fraction with γ mobility and the green tinge characteristic for particles rich in PL. The applied buffer, which we had previously used in our study with phosphatidylcholine liposomes and γ-mobility lipoproteins [28], enabled clear differentiation of particles with low electrophoretic mobility. A clear demonstration of the presence of LpX was also presented by Inamoto et al. [29], who applied cholesterol and triglyceride staining following lipoprotein electrophoresis. Other electrophoretic techniques have also been used to detect LpX, for example nondenaturing polyacrylamide gradient gel electrophoresis revealed the presence of LpX as particles in the intermediate region of size and with a narrower range of sizes than VLDL [3]. In Quantimetrix Lipoprint LDL subfraction analysis, LpX was detected as a large band between the loading and separation gels, since LpX particles are too large to enter 3% polyacrylamide gel [25]. Taking into account our report as well as those of others, it can be concluded that the presence of LpX manifests in different ways in electrophoresis, in a patient, and in an applied electrophoresis set dependent manner. Thus, the agarose electrophoresis results obtained in routine laboratory testing concerning patients with severe hypercholesterolaemia should be analysed very carefully. Eventually, other electrophoretic techniques or methods, such as ultracentrifugation, nuclear magnetic resonance spectroscopy, and immunological analysis, may be useful for LpX detection [6]. Unfortunately, these methods are usually available only in specialised laboratories, reducing the potential for their use in routine practice. However, it has been shown that the presence of LpX can also be indicated using biochemical tests such as FC and PL levels, as well as FC:CE and TC/apoB ratios. Among these, the most readily available in routine practice seems to be the last named [6]. LpX contains cholesterol but not apoB; thus, TC/apoB ratios are increased in patients with LpX. Reference values for TC/apoB ratios have been developed, and significantly increased TC/apoB ratios have been observed in many patients with LpX [13] [19] [22] [25] [26] [27] [30]. As well, in our patient the TC/apoB ratio was significantly increased. However, an only slightly increased TC/apoB ratio may be related to the co-existence of LpX with LDL, which occurs in some patients [7]. In these patients, the apoB level is increased, lowering the TC/apoB ratio, sometimes even to established reference values [6], as observed in the patient with LpX described by Sivakumar et al. [21]. Thus, it can be concluded that TC/apoB ratios, along with other biochemical analyses such as FC:CE ratio, do not always directly indicate the presence of LpX, making the detection of this lipoprotein difficult.

Thus, taking into account the heterogeneity of laboratory manifestations of the presence of LpX in plasma, we conclude that, in routine practice, any abnormalities in electrophoresis or discrepancies between electrophoresis and lipid-related biochemical tests (i.e. between electrophoresis and TG level or between TC and apoB levels) should be analysed very carefully and should raise suspicions of the presence of LpX, especially in hypercholesterolaemic patients with cholestasis.

### Ethics

All procedures were in accordance with the ethical standards of the Helsinki declaration.

The analysis was performed using material obtained from the patient during hospitalization for diagnostic purposes, and informed consent was previously given for the scope of treatment. The patient gave oral consent to publication in the presence of two witnesses. Due to the patient's death, written informed consent to publication has been obtained from the closest relative, the patient's mother.

*Acknowledgements*. This work was supported by the Medical University of Gdansk grants no. ST 02- 0125/07/524 and ST 02-0085/07/182.

## Conflict of interest statement

The authors stated that they have no conflicts of interest regarding the publication of this article.

## List of abbreviations

ApoAI, apolipoprotein AI; ApoB, apolipoprotein B; ALT, alanine aminotransferase; ALP, alkaline phosphatase; AST, aspartate aminotransferase; CE, cholesterylester; CVD, cardiovascular disease; FC, free cholesterol; GGTP, gamma-glutamyl transferase; HDL, high density lipoprotein; HL, hepatic lipase; Hlp, hyperlipoproteinaemia; HoFH, homozygous familial hypercholesterolaemia; LA, lipoprotein apheresis; LCAT, lecithin:cholesterol acyltransferase; LDL, low density lipoprotein; LTx, liver transplantation; LpX, lipoprotein X; PL, phospholipid; TC, total cholesterol; TG, triglyceride.

## References

[1] Cuchel M, Bruckert E, Ginsberg H N, Raal F J, Santos R D, Hegele R A, Kuivenhoven J A, Nordestgaard B G, Descamps O S, Steinhagen-Thiessen E, Tybjaerg-Hansen A, Watts G F, Averna M, Boileau C, Boren J, Catapano A L, Defesche J C (2014). Homozygous familial hypercholesterolaemia: New insights and guidance for clinicians to improve detection and clinical management: A position paper from the Consensus Panel on Familial Hypercholesterolaemia of the European Atherosclerosis Society. Eur Heart J.

[2] Jankowski K, Wyzgał A, Wierzbicka A, Tronina O, Durlik M, Pruszczyk P (2015). Rapid normalization of severe hypercholesterolemia mediated by lipoprotein X after liver transplantation in a patient with cholestasis: A case report. Acta Biochim Pol.

[3] Phatlhane D V, Zemlin A E (2015). Severe hypercholesterolemia mediated by lipoprotein X in a patient with cholestasis. Ann Hepatol.

[4] Turchin A, Wiebe D A, Seely E W, Graham T, Longo W, Soiffer R (2005). Severe hypercholesterolemia mediated by lipoprotein X in patients with chronic graft-versus-host disease of the liver. Bone Marrow Transplant.

[5] Soros P, Bottcher J, Maschek H, Selberg O, Muller M J (1998). Lipoprotein-X in patients with cirrhosis: Its relationship to cholestasis and hypercholesterolemia. Hepatology.

[6] Crook M A (2013). Lipoprotein X: Clinical implications. Ann Clin Biochem.

[7] Jahn C E, Schaefer E J, Taam L A, Hoofnagle J H, Lindgren F T, Albers J J (1985). Lipoprotein abnormalities in primary biliary cirrhosis: Association with hepatic lipase inhibition as well as altered cholesterol esterification. Gastroenterology.

[8] Chang P Y, Lu S C, Su T C, Chou S F, Huang W H, Morrisett J D, Chen C, Liau C, Lee Y (2004). Lipoprotein-X reduces LDL atherogenicity in primary biliary cirrhosis by preventing LDL oxidation. J Lipid Res.

[9] Longo M, Crosignani A, Battezzati P M, Squarcia G C, Invernizzi P, Zuin M, Podda M (2002). Hyperlipidaemic state and cardiovascular risk in primary biliary cirrhosis. Gut.

[10] Ossoli A, Neufeld E B, Thacker S G, Vaisman B, Pryor M, Freeman L A, Brantner C A, Baranova I, Francone N O, Demosky S J, Vitali C, Locatelli M, Abbate M, Zoja C (2016). Lipoprotein X Causes Renal Disease in LCAT Deficiency. PLoS One.

[11] Neely G R D, Boot C S (2017). Laboratory investigation of lipo - protein X. Clin Lipidol.

[12] Fellin R, Manzato E (2019). Lipoprotein-X fifty years after its original discovery. Nutr Metab Cardiovasc Dis.

[13] Brandt E J, Regnier S M, Leung E K, Chou S H, Baron B W, te Helen S, Davidson M H, Sargis R M (2015). Management of lipoprotein X and its complications in a patient with primary sclerosing cholangitis. Clin Lipidol.

[14] Yehya A, Huang R, Bernard D W, Gotto A, Robbins R J (2018). Extreme hypercholesterolemia in cholestatic sarcoidosis due to lipoprotein X: Exploring the cholesterol gap. J Clin Transl Endocrinol Case Rep.

[15] Sorokin A, Brown J L, Thompson P D (2007). Primary biliary cirrhosis, hyperlipidemia, and atherosclerotic risk: A systematic review. Atherosclerosis.

[16] Rosenson R S, Baker A L, Chow M J, Hay R V (1990). Hyperviscosity Syndrome in a Hypercholesterolemic Patient with Primary Biliary Cirrhosis. Gastroenterology.

[17] Heinl R E, Tennant H M, Ricketts J C, Rice C R, Robinson C B, Sandesara P B, Moriarty P M, Sperling L (2017). Lipoprotein-X disease in the setting of severe cholestatic hepatobiliary autoimmune disease. J Clin Lipidol.

[18] Matsushima K, Sugiuchi H, Anraku K, Nishimura H, Manabe M, Ikeda K, Ando Y, Kondo Y, Ishitsuka Y, Irikura M, Irie T (2015). Differences in reaction specificity toward lipoprotein X and abnormal LDL among 6 homogeneous assays for LDL-cholesterol. Clin Chim Acta.

[19] Benjamini Y, Hochberg Y (1995). Controlling the False Discovery Rate: A Practical and Powerful Approach to Multiple Testing. Journal of the Royal Statistical Society: Series B (Methodological).

[20] Futatsugi A, Hidaka E, Kubota N, Nishijima F, Yoshizawa K, Ishimine N (2015). Abnormal Serum Total Protein Measurement by Lipoprotein-X in an Infant with Biliary Atresia. Rinsho Byori.

[21] Sivakumar T, Chaidarun S, Lee H K, Cervinski M, Comi R (2011). Multiple lipoprotein and electrolyte laboratory artifacts caused by lipoprotein X in obstructive biliary cholestasis secondary to pancreatic cancer. J Clin Lipidol.

[22] Hussain I, Ahmad Z, Garg A (2015). Extreme hypercholesterolemia presenting with pseudohyponatremia: A case report and review of the literature. J Clin Lipidol.

[23] Farooqi M S, Hashim I A (2015). A Woman with Primary Biliary Cirrhosis and Hyponatremia. Clin Chem.

[24] Ravella S, Lefavour G S, Carayannopoulos M O, Parikh A (2015). The Case đ Hyponatremia in a patient with obstructive jaundice. Kidney Int.

[25] Foley K F, Silveira M G, Hornseth J M, Lindor K D, McConnell J P (2009). A Patient with Primary Biliary Cirrhosis and Elevated LDL Cholesterol. Clin Chem.

[26] Chow A, Rifici V A, Schneider S H (2016). Lipoprotein-X in a Patient with Lymphoplasmacytic Sclerosing Cholangitis: An Unusual Cause of Secondary Hypercholesterolemia. AACE Clin Case Rep.

[27] Stepien K M, Divyateja H, Ahmed F, Prinsloo P, Gupta P (2013). Lipoprotein X in a patient with cholestasis and hypertriglyceridaemia. Ann Clin Biochem.

[28] Ćwiklińska A, Kortas-Stempak B, Gliwińska A, Pacanis A, Kuchta A, Wróblewska M (2014). Interaction Between VLDL and Phosphatidylcholine Liposomes Generates New g-LpE-like Particles. Lipids.

[29] Inamoto Y, Teramoto T, Shirai K, Tsukamoto H, Sanda T, Miyamura K, Yamamori I, Hirabayashi N, Kodera Y (2005). Severe Hypercholesterolemia Associated with Decreased Hepatic Triglyceride Lipase Activity and Pseudohyponatremia in Patients after Allogeneic Stem Cell Transplantation. Int J Hematol.

[30] Suzuki L, Hirayama S, Fukui M, Sasaki M, Hiroi S, Ayaori M, Terai S, Tozuka M, Watada H, Miida T (2017). Lipoprotein-X in cholestatic patients causes xanthomas and promotes foam cell formation in human macrophages. J Clin Lipidol.

